# Neuro–Immune Interactions in Severe COVID-19 Infection

**DOI:** 10.3390/pathogens11111256

**Published:** 2022-10-29

**Authors:** Elena Rossi, Luciano Mutti, Andrea Morrione, Antonio Giordano

**Affiliations:** 1 Department of Medicine, Surgery and Neuroscience, University of Siena, 53100 Siena, Italy; 2 Sbarro Institute for Cancer Research and Molecular Medicine, Center for Biotechnology, Department of Biology, College of Science and Technology, Temple University, Philadelphia, PA 19122, USA; 3 Italian Group for Research and Therapy for Mesothelioma (GIMe), 27058 Voghera, Italy; 4 Department of Biotechnological and Applied Clinical Sciences, University of L’Aquila, Via Vetoio, Coppito 2, 67100 L’Aquila, Italy

**Keywords:** severe COVID-19, neurologic symptoms, renin-angiotensin system, inflammatory responses, lymphopenia, neuro–immune interactions, aging

## Abstract

SARS-CoV-2 is a new coronavirus that has affected the world since 2019. Interstitial pneumonia is the most common clinical presentation, but additional symptoms have been reported, including neurological manifestations. Severe forms of infection, especially in elderly patients, present as an excessive inflammatory response called “cytokine storm”, which can lead to acute respiratory distress syndrome (ARDS), multiorgan failure and death. Little is known about the relationship between symptoms and clinical outcomes or the characteristics of virus–host interactions. The aim of this narrative review is to highlight possible links between neurological involvement and respiratory damage mediated by pathological inflammatory pathways in SARS-CoV-2 infection. We will focus on neuro–immune interactions and age-related immunity decline and discuss some pathological mechanisms that contribute to negative outcomes in COVID-19 patients. Furthermore, we will describe available therapeutic strategies and their effects on COVID-19 neurological symptoms.

## 1. Introduction

Coronaviruses are enveloped viruses with a plus-strand RNA genome identified in several mammals and humans [1] that can be classified in four genera: alfa, beta, gamma and delta [2]. The most common coronaviruses infect humans and are associated with mild symptoms; however, some coronaviruses can cause severe pneumonia and life-threating clinical conditions, as was the case with SARS [3,4] and MERS [4] in the past.

SARS-CoV-2 is a novel and highly pathogenic betacoronavirus first reported in December 2019 in Wuhan, China, and the cause of the global COVID-19 pandemic.

Fever, cough and myalgia are common symptoms at onset of illness, whereas some patients with severe COVID-19 develop acute respiratory distress syndrome (ARDS), thereby requiring intensive care unit (ICU) admission and oxygen therapy [5]. However, the clinical presentation of SARS-CoV-2 infections is quite variable [6,7], and some patients have developed both mild and severe neurological symptoms [8,9].

This variability of COVID-19 clinical manifestations could be explained by the ubiquitous expression of human cell receptors with which viral spike 1 protein can interact.

Angiotensin-converting enzyme 2 (ACE2) [10] and transmembrane protease serine 2 (TMPRSS2) are the main SARS-CoV-2 entry receptors [11,12]; they are expressed not only in the lower respiratory tract (most on type II pneumocytes) but also in extrapulmonary sites, such as the heart, kidney, endothelial cells of blood vessels and the nervous system [13,14].

These observations have sparked interest in the ability of the virus to interact with different tissues and damage them directly or indirectly through inflammatory responses.

The increase in angiotensin II levels [15] consequent to the dysregulation of the renin-angiotensin system (RAS), as well as the significant release of proinflammatory cytokines, highlights the ability of SARS-CoV-2 to enhance inflammatory responses, which might lead to lung damage and neurological consequences in severe forms of COVID-19.

Severe forms of SARS-CoV-2 infection result in an increase in inflammatory responses, likely due to immune system dysregulation. Furthermore, the decrease in immune competence related to older age contributes to the development of worse outcomes [16]. The overall case-fatality rate (the probability of dying after developing symptoms) is more elevated in older people infected with COVID-19 than younger patients [17,18].

Although previous coronaviruses SARS and MERS caused epidemic diseases, they did not have the ability to spread worldwide like SARS-CoV-2 [19,20], although the reasons for this difference are not completely understood. However, some differences among coronaviruses can explain this discrepancy. The binding affinity of SARS-CoV-2 S protein is higher than that of SARS-CoV S protein, conferring more efficient infectiousness [21]. SARS-CoV-2 actively replicates in the upper respiratory tract in the early stage of infection, determining a high transmission rate in asymptomatic and mildly symptomatic patients [21,22]. Conversely, SARS and MERS had a tropism for lower airways, and the peak of viral shedding occurred after one week of symptom onset, providing more time to isolate cases before human-to-human transmission [23]. The majority of SARS and MERS outbreaks occurred through nosocomial transmission [4,24]. These characteristics provide a likely explanation for the distinct pattern of spread of SARS and MERS compared to that of SARS-CoV-2 infection.

Various therapeutic strategies have been tested since the beginning of the pandemic; however, owing to patient characteristics, comorbidities and differences in clinical courses, results have been somewhat controversial.

In this narrative review, we will provide an overview of the principal therapeutic approaches used to date and their effects on COVID-19 neurologic symptoms.

## 2. Nervous System and COVID-19

Although respiratory symptoms are the main clinical manifestations of SARS-CoV-2 infection, neurological involvement is clearly present [25,26]. Anosmia (loss of smell), ageusia (loss of taste) [27,28], cough, fatigue and headache are reported as the most common mild neurological symptoms, and some patients can experience severe neurological symtoms, such as encephalopathy and acute cerebrovascular diseases [25].

### 2.1. Neurological Symptoms and COVID-19

Among 404 COVID-19 patients involved in a clinical study, 51.5% presented with central nervous system (CNS) symptoms [29] (Table 1). In a retrospective case series of 214 infected patients, 36.4% had mild and severe neurologic manifestations, including impaired consciousness and cerebrovascular diseases [8]. Furthermore, data collected through an online network of case report notification portals across major UK neuroscience bodies reported that among 125 COVID-19 patients, 31% presented with acute alteration in mental status, encephalopathy or encephalitis, whereas 62% had a cerebrovascular event [30]. In a cohort of 43 SARS-CoV-2-infected patients, 12 manifested inflammatory CNS syndromes, and 8 had ischaemic stroke [31].

Among 841 hospitalized COVID-19 patients, 57.4% developed neurologic symptoms, which were non-specific in the early stage and severe in the later stages of the infection [32]. Notably, the majority of cases presenting with altered consciousness were associated with hypoxemia and older age and were related to the severity of the disease [32]. In contrast, histopathological observations in 19 human ACE2-expressing transgenic mice infected with SARS-CoV-2 revealed interstitial pneumonia with thickened alveolar septa and infiltration of lymphocytes, whereas in other organs, including brain, there were no histopathological changes or viral antigens [33].

However, new onset of multiple demyelinating lesions was found by MRI in a case report of a COVID-19 patient [34]. Neuroimaging analysis highlighted other brain abnormalities in patients infected by the new coronavirus, such as intracranial haemorrhages, stroke and encephalitis [35,36,37]. Additionally, the virus was detected in neural and capillary endothelial cells during post-mortem brain examinations in a COVID-19 patient [38].

A case report described a 74-year-old patient with COVID-19 encephalopathy, suggesting that age, pre-existing conditions and acute respiratory symptoms increased the risk of encephalopathy upon initial presentation [39]. On the other hand, a retrospective study showed that patients with respiratory symptoms experienced more severe disease and higher mortality risk than those who presented with only neurologic symptoms, suggesting an independent course of organ involvement [40]. However, the finding of neural-tissue-damage markers significantly increased in the blood of severe COVID-19 patients is either an interesting possible predictor of clinical outcome or further evidence of neurological involvement in the pathogenicity of the disease [41,42,43]. Analysis of CSF in a cross-sectional study showed that patients with COVID-19 had higher levels of CSF neurofilament chain compared to controls, and patients with neurological symptoms exhibited a more marked immune activation biomarker profile [44].

Recently, an in vivo and in vitro study analysed the neuropathological potential of SARS-CoV-2 variants, reporting that Delta and Omicron BA.1 variants had a reduced neurotropic and neurovirulent potential compared to the previous D614G variant [45].

Neurological symptoms are not only present during COVID-19 infection but can persist as post-COVID syndrome. Chronic cough associated with fatigue, asthenia, chronic pain and cognitive impairment can be present in healed patients [46,47].

Moreover, cough, myalgia, fatigue and headache are not specific to coronavirus diseases but also occur in other upper respiratory tract infections, including influenza [48,49].

Based on these data, neurologic symptoms are one of the clinical aspects of SARS-CoV-2 infection and are prevalently associated with conditions in which the immune system is diminished, such as in elderly patients and severe clinical profiles. Neurological manifestations are associated with more severe disease, as supported by a longer recovery, higher death rate during hospitalization and the presence of sequelae at discharge [50]. In addition, the decrease in pO2 levels associated with lung damage can lead to severe hypoxia, which contributes to brain damage. Thus, neurological symptoms, elevated inflammatory responses and hypoxia could increase the risk of developing a severe clinical course of COVID-19.

### 2.2. COVID-19 and Possible Routes to CNS

**Figure 1:**
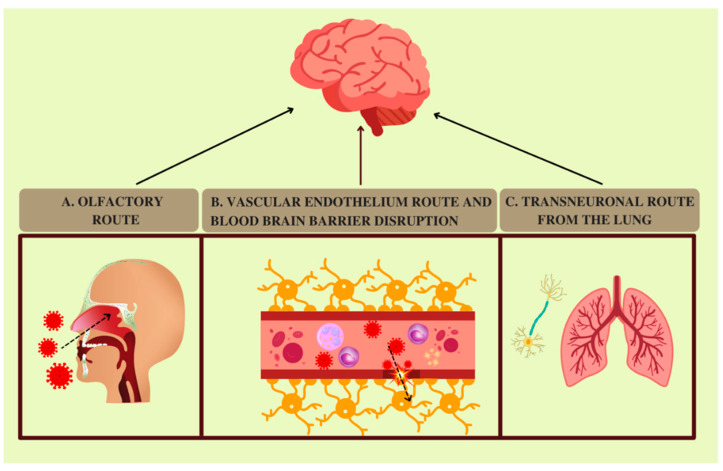
COVID-19 and potential routes to the CNS. (**A**) Olfactory route. SARS-CoV-2 may reach the cribriform plate and olfactory bulb through olfactory nerve projections and gain access to the brain [53,54]. This hypothesis is supported by the presence of SARS-CoV-2 RNA in olfactory mucosa and the neuroanatomical area receiving olfactory tract projections [55]. (**B**) Vascular endothelium route and blood–brain barrier disruption. Another possible viral route to the nervous system starts from the interaction between SARS-CoV-2 and the vascular endothelium expressing ACE2. The binding of SARS-CoV-2 to ACE2 can induce an increase in vascular permeability and blood–brain barrier disruption [62]. In addition, cytokine and inflammatory mediators transported through the circulatory system might affect the integrity of the blood–brain barrier [63,64]. (**C**) Transneuronal route from the lungs involves a potential SARS-CoV-2 neuronal retrograde dissemination from the lungs. Because the vagal nerve innervates lungs, the virus may use it to gain access into the brain [70,71], as SARS-CoV-2 has been detected in the vagal nerve fibres [72].

Coronaviruses are mainly respiratory viruses, but their neuropathological potential is well known [51,52]. Although some patients exhibit neurological symptoms during COVID-19, it has not been established whether the virus can reach the CNS. Thus, in this section, we discuss some hypothesis regarding SARS-CoV-2 routes to the nervous system (Figure 1).

As anosmia and ageusia are associated with SARS-CoV-2 infection, SARS-CoV-2 may reach the CNS from olfactory nerve projections into the nasal cavity and further to the cribriform plate and olfactory bulb [53,54]. The possibility of axonal transport is supported by the detection of SARS-CoV-2 RNA in the olfactory mucosa and in neuroanatomical areas receiving olfactory tract projections [55]. A similar route leading to a rapid, trans-neuronal spreading to a connected area of the brain was investigated in SARS-CoV infection [56].

Furthermore, high expression of viral receptor ACE2 in nasal epithelial cells (goblet and ciliate cells) [57] and non-neuronal cells of the olfactory epithelium suggests the hypothesis that the infection of these non-neuronal cells initiates an inflammatory response, which impairs olfactory neuronal function [58].

Analysis on human olfactory epithelia from autopsies of infected patients showed the presence of SARS-CoV-2 RNA in the non-neuronal layers of the olfactory epithelium and the downregulation of olfactory receptors and olfactory receptor-signalling genes [59].

A recent multicentre postmortem cohort study showed that COVID-19 patients had worse olfactory axonal damage and microvascular pathology compared to controls, although these abnormalities did not appear to be associated with disease severity [60].

Notably, intranasal inoculation of SARS-CoV-2 Alpha, Beta, Delta and Omicron variants in transgenic mouse models expressing human ACE2 showed that all variants except Omicron can lead to infection and virus spread to the brain within 5 to 7 days [61].

Another potential viral route to the CNS starts from the interaction between SARS-CoV-2 and the vascular endothelium expressing ACE2. SARS-CoV-2 binds its receptor and can induce an increase in vascular permeability and blood–brain barrier (BBB) disruption [62].

Both viral components and molecules linked with viral activities, such as cytokines and toxic metabolites, are transported through the circulatory system and may affect the integrity of the blood–brain barrier [63]. The detection of anti-SARS-CoV-2 antibodies in the CSF of encephalopathic COVID-19 patients suggests the release of inflammatory mediators into the CNS associated with BBB disruption, supporting a pattern of neuroinflammation and neurodegeneration [64]. Moreover, the discovery of altered brain vasculature associated with SARS-CoV-2 neuroinvasion in animal models, as well as the presence of ischaemic damage in COVID-19 human brain autopsies, led to the hypothesis that neuroinvasion, local hypoxia, impaired vasculature and disruption of the BBB can enhance the vulnerability of the brain to viral infection [65].

Interesting data have indicated that SARS-CoV-2 S1 protein was able to cross the murine BBB and spread into the brain, although these results were not confirmed in an in vitro model of the human BBB [66]. Furthermore, a recent study reported that the entry and replication of SARS-CoV-2 occurred after infecting human induced pluripotent stem cell-derived brain capillary endothelial-like cells [67].

However, analysis of CSF of COVID-19 patients with neurological symptoms revealed an abnormal presence of macrophages and signs of BBB leakage, suggesting a possible indirect mechanism of damage due to microglia activation and proinflammatory cytokine release [68].

A transneuronal route from the lung is another possible means of viral entry to the CNS.

Some coronaviruses reach medullary cardiorespiratory centres from mechanoreceptors and chemoreceptors in lower respiratory airways and synapse-connected routes [69]. Because the vagal nerve innervates lungs, SARS-CoV-2 might use it to gain access to the nucleus of the solitary tract and nucleus ambiguous, suggesting a role of the brainstem in respiratory failure [70,71]. An interesting study detected SARS-CoV-2 in the vagal nerve fibres [72]. Moreover, immunohistochemical analysis found SARS-CoV-2 proteins in cranial nerves, such as vagal nerves; however, the presence of the virus in the CNS was not associated with severity of neuropathological changes [73]. However, the detection of interactions between the inflammatory and immune cell factors and the sensory neuronal innervation of the lung revealed potential neuro–immune interactions, which might contribute to important aspects of COVID-19 disease severity, including acute distress respiratory syndrome [74].

## 3. Renin-Angiotensin System (RAS) and COVID-19-Related Neurologic Involvement

The renin-angiotensin system is a fundamental system with respect to the regulation of blood pressure. ACE converts angiotensin I to angiotensin II (Ang II), whereas ACE2 transforms Ang II to angiotensin 1–7. Ang II is a strong vasoconstrictor and participates in several proinflammatory signals, whereas angiotensin 1–7 acts as a vasodilatory and anti-inflammatory agent.

Notably, Ang II binds AT_1_ receptors and contributes to several proinflammatory responses, such as leukocyte recruitment, stimulation of innate immune response and increase in reactive oxygen species (ROS) production [75].

The binding of SARS-COV-2 to ACE2 leads to ACE2 downregulation from the cell surface, shifting the renin-angiotensin system into proinflammatory mode and triggering ROS production, fibrosis and proinflammatory cytokine release, especially in the lungs [76,77,78]. Accordingly, a study on *ACE2* knockout mice demonstrated that ACE2 downregulation is linked to increased production of Ang II or worsening of acute lung injury [79]. Moreover, angiotensin II levels were found to be markedly increased and associated with viral load and lung damage in plasma samples of COVID-19-infected patients [15].

The metalloproteases ADAM17 was reported to degrade ACE2 by shedding it from the cell membrane into serum [80]. Angiotensin II upregulated ADAM17, the expression of which increases with age [80]. An in vitro study demonstrated that soluble ACE2 could interact with the spike protein of SARS-CoV-2, thereby forming a complex capable of entering cells through receptor-mediated endocytosis via the AT1 receptor [81]. Moreover, the level of soluble ACE2 is associated with the severity of COVID-19 [82].

Recent findings revealed increased levels of extracellular vesicles expressing ACE2 in plasma of COVID-19 patients and that engineered ACE2 extracellular vesicles blocked SARS-CoV-2 infection by competing with cellular ACE2, thereby suggesting a potential innate antiviral mechanism [83].

Notably, SARS-CoV-2 variants show different binding affinity to ACE2 receptors. A recent computational study found that the Omicron variant had higher affinity to human ACE2 than the Delta strain, suggesting an increased potential for transmission [84]. Nevertheless, further analysis of the differences in viral spread among SARS-CoV-2 variants and the role of ACE2 receptor is required.

The finding of ACE2 receptor expression in different areas and cell types of the brain suggests new possible scenarios in SARS-CoV-2 pathogenesis [85]. Analysis of ACE2 expression in 85 human tissues using multiple tissue expression arrays showed significant ACE2 expression in different brain regions, particularly in the pons and in the medulla oblongata [86]. A recent study demonstrated SARS-CoV-2 infection in neuronal cells derived from human ACE2-expressing mice, which resulted in upregulation of genes involved in inflammation and innate immunity [87].

An intriguing study of human brain organoids revealed that even a low level of ACE2 is sufficient for viral entry into neurons [88]. In addition, ACE2 depletion and its consequences could play a role in cerebrovascular events in COVID-19 patients [89].

The finding of ACE2 expression in mature choroid plexus cells but not in neurons suggests the ability of SARS-CoV-2 to infect choroid plexus epithelial cells, leading to a disruption of barrier integrity [90]. Conversely, a recent study showed ACE2 expression in human pluripotent stem-cell-derived mixed neurons via immunocytochemistry, supporting the neuroinvasive potential of SARS-CoV-2 [91].

However, the impairment of the BBB endothelial lining might be triggered either by SARS-CoV-2 binding on the ACE2 receptor or by immune invasion with the same goal of gaining entry into the brain [92].

The discovery of angiotensin II receptor expression in neurons and in circumventricular organs suggests the existence of an endogenous brain angiotensin II system integrated with the peripheral system [93]. Accordingly, ACE2 dysfunction and the increase in angiotensin II levels were proposed as parts of the pathogenetic mechanism responsible for intracerebral haemorrhage in a 79-year-old COVID-19 patient [94].

ACE2 may play a protective role in the brain by balancing the ACE/Ang II axis [95]. Angiotensin 1–7 reduces inflammation, oxidative stress and neuronal apoptosis in the brain [96]. In addition, the dorsal vagal complex, which is involved in both cardiorespiratory regulation and inflammatory reflex, is an Ang II target [97].

Notably, the dysregulation of RAS and the immune system contributes to the creation of a hyperinflammatory environment during severe forms of infection. The finding of *ACE2* as a human interferon-stimulated gene and the upregulation of ACE2 by type I INF suggests that SARS-CoV-2 could use species-specific interferon-driven upregulation of ACE2 to enhance infection [98]. Nevertheless, a low and delayed interferon response found in the late phase of critical COVID-19 disease [99] may alter ACE2 expression and function, increasing angiotensin II levels and enhancing inflammation.

## 4. Immune System and COVID-19-Related Neurologic Involvement

### 4.1. Type I Interferon and COVID-19

Type I interferon (INF) is strongly released during viral infections either to inhibit viral replication or to improve immune responses.

The interference of INF production by SARS-CoV-2-encoded proteins can allow viral replication in the host cells at the early stage of infection and lead to a delayed type I INF response in COVID-19 patients [100]. An interesting study proposed type I INF deficiency as a hallmark of severe SARS-CoV-2 infection, which was supported by the identification of an impaired type I INF response associated with high blood viral load, increased TNFα/IL6 levels and high neutrophilia [101]. In contrast, another study reported that type I INF signalling, together with TNF and IL1B responses, was upregulated in severe COVID-19 cases, thereby contributing to the progression of hyperinflammatory response [102]. However, the finding of high level of INFs in lower airways of patients with severe disease and the upper airways of patients with mild symptoms suggests a link between INF timing and anatomical sites of production and varied clinical outcomes [103].

The discovery of interferon-stimulated genes with antiviral activity in different neuronal subtypes could influence viral spread through the CNS [104], opening new scenarios for neuro–immune interactions in COVID-19 pathogenesis. Moreover, a recent study reported upregulation of interferon signalling pathways in the neurovascular unit of COVID-19 patients [67].

An interesting study demonstrated that severe neuro-COVID (term to define COVID-19 patients with neurological sequelae) showed a broad clonal T-cell expansion and curtailed INF response compared to mild neuro-COVID [105]. Notably, as INFs promote T-cell survival and action, delayed INF response can inhibit T-cell proliferation or cause their functional exhaustion [106], underlying the importance of the T cell/INF relationship with respect to the severity of COVID-19 disease.

### 4.2. Macrophages/Microglia and COVID-19

Severe acute respiratory syndrome effects and hyperinflammatory responses are associated with SARS-CoV-2 disease progression and complications [107]. COVID-19 impairs the immune system in severe cases either by replacing alveolar macrophages with recruited inflammatory cells or by infecting epithelial and macrophage subsets [108]. Moreover, myeloid cells and subsets of inflammatory macrophages [109] differ in transcriptional signatures between blood and airway compartments, underlying a complex model of local and systemic host response to the new coronavirus [110]. Thus, the link between inflammatory responses and different effects on the nervous system needs to be further elucidated. Systemic hypoxia, inflammation and oxidative stress associated with SARS-CoV-2 lung injury can have a detrimental effect on the nervous system [111], supporting a possible integrated and bidirectional connection between the lungs and brain [112,113].

Interactions between microglia, CNS macrophages and the virus or peripheral inflammatory signals of innate immune cells might play a role in the neuropathogenesis of COVID-19. The “two-hit” hypothesis, which proposes priming of microglia by aging, stress or chronic low-grade inflammatory diseases followed by their hyperactivated inflammatory reaction to secondary immune stimuli, such as SARS-CoV-2, can explain disease severity in some patients [114]. Recently, intranasal virus inoculation in mouse models revealed microglial activation and microgliosis on days 6 and 7 post infection only in mice infected with the Delta variant with brain involvement but not in those infected with the Omicron variant [61].

Viral proteins and molecular complexes from damaged cells could enter the brain through a damaged BBB, then act as pathogen-associated molecular patterns (PAMPs) and damage-associated molecular patterns (DAMPs) to activate an innate immune response in microglia [115].

SARS-CoV-2 binding to ACE2 receptor could promote microgliosis, astrogliosis and increased BBB permeability, which would allow immune cells to infiltrate different CNS regions [116]. Extensive inflammation (clustering of microglia, sparse T lymphocytes and neutrophil plugs) was detected in the medulla oblongata and olfactory bulb in COVID-19 autopsies [117]. Furthermore, ACE2 downregulation induced by SARS-CoV-2 may alter the brain RAS system by hyperactivating Ang II/AT1 receptor proinflammatory signalling on microglia and reducing antioxidant activity of angiotensin 1–7 [118]. These data support a link between a dysregulated renin-angiotensin system and glia behaviour during COVID-19 infection.

Analysis of bronchoalveolar lavage fluid of critical COVID-19 patients revealed an abundance of macrophages and neutrophils, as well as high levels of inflammatory cytokines [119]. In particular, the increase in the C-C motif chemokine receptor 2 (CCR2) signal, which promotes chemotaxis of monocytes and macrophages toward inflammatory sites and the high concentration of its ligand monocyte, chemoattractant protein-1 (CCL2/MCP1), is associated with more severe disease [101,120]. Monocyte chemoattractant protein-1 (MCP1) has the ability to alter the expression and distribution of some tight-junction-associated proteins in brain vascular endothelial cells [121]. Thus, high levels of MCP1 are observed in severe cases of COVID-19, although their role in the alteration of the BBB and viral spread needs to be elucidated. Moreover, a study demonstrated that SARS-CoV-2 spike protein induced a loss of BBB integrity and triggered a proinflammatory response on brain endothelial cells [122]. Thus, the inflammatory and immune mediators trafficking between the lungs and brain may play a role in COVID-19 infection severity.

Alveolar macrophages contribute to the resolution of inflammation through various mechanisms, such as the secretion of anti-inflammatory/repair factors, the removal of dying cells and the promotion of regulatory T-cell differentiation [123]. Alveolar macrophages are helped by another lung-resident cell population called nerve- and airway-associated macrophages (NAMs), which are in close contact with vagal fibres innervating the airways [124,125]. Alveolar macrophages deal with viral clearance, whereas NAMs contribute to the regulation of lung inflammation [124]. Thus, NAM depletion leads to increased inflammatory cytokine production and innate immune cell infiltration [124]. An intriguing hypothesis is that NAMs act in combination with neuronal cells to moderate inflammation and that dysregulation of this system could contribute to a cytokine storm and ARDS in patients with severe COVID-19 [125].

In contrast, a study demonstrated that the distribution of SARS-CoV-2 RNA within tissues is not linearly linked to the presence or the nature of the inflammatory response, especially in the lungs [126]. However, in some patients, a dysfunctional immune response [127,128,129] occurs, which triggers a cytokine storm mainly driven by IL-2, IL-6, IL-7, IL-10, IP-10, MIP-1α and MCP1 [130,131].

The risk of indirect neurological symptoms may be increased by cytokines through widespread organ damage [132]. Specifically, some of these proinflammatory cytokines can either increase BBB permeability and its disruption or further activate glial cells, supporting the possibility of deleterious crosstalk between the brain and the immune system [133].

### 4.3. Neutrophils and COVID-19

Neutrophils are implicated in the pathogenesis of COVID-19 infection, as demonstrated by the presence of neutrophil infiltration in pulmonary capillaries and their extravasation into the alveolar spaces in autopsy samples [134]. Analysis of the relationship between lung injury and leukocyte counts revealed increased neutrophil counts parallel to the CT value of lesions in severe cases of hospitalized COVID-19 patients [135].

Analysis of granulocyte samples from COVID-19 patients revealed either an enrichment of signatures typical of immature neutrophils or evidence of both inflammatory and suppressive features, underlying a peripheric dysregulation in conjunction with an alteration in the cell transcriptional program [136]. Notably, the neutrophil-to-lymphocyte ratio (NLR) was identified as one of the most important prognostic factors for critical illness in patients with SARS-CoV-2 infection [137].

NETs (neutrophil extracellular traps) released by neutrophils are another immune weapon against pathogens. However, excessive quantities of NETs are deleterious in some cases. The release of hyperactivated neutrophils and NETs not only contributes to lung damage but also promotes systemic inflammation in severe SARS-CoV-2 infection [138], although the systemic presence of NETs is likely not associated with the formation of NETs in peripheral organs [139].

The increase in NETs correlates with severity of respiratory illness in COVID-19 patients [140]. The release of NETs and neutrophil infiltration drive necroinflammation in coronavirus infections [141]. The presence of cytotoxic proteins carried by NETs may directly damage the BBB, whereas NETs released by extravasated granulocytes could activate microglia [142,143].

NETs can communicate with macrophages [134] and interact with T, NK and B cells to support the progression of disease [135]. Furthermore, an interesting study demonstrated that viable SARS-CoV-2 increased the release of NETs by neutrophils via mechanisms dependent on the ACE2–serine protease axis [144]. Thus, we hypothesize an indirect role of neutrophils in the pathogenesis of neurological symptoms in COVID-19, likely mediated by the release of inflammatory factors and interactions with other immune cells.

### 4.4. CD4 and CD8 T Cells and COVID-19

Lymphopenia is a common feature in patients with severe COVID-19, and the presence of upregulated exhaustion markers on cytotoxic lymphocytes is representative of adaptive immune system impairment [145,146,147]. Recently, a study demonstrated that T-cell apoptosis is associated with T-cell lymphopenia in COVID-19 patients [148]. Moreover, a retrospective study reported that the presence of both CD4 and CD8 T cells, as well as regulatory T cells, was decreased in severe cases [149]. Other recent data show that Omicron spike mutations occurred more commonly in regions frequently targeted by CD8 T cells [150]. Thus, activated phenotypes of CD8 T cells could be used to predict clinical outcomes in COVID-19 patients [151].

COVID-19 can damage CD4 T-cell functions and promote excessive activation and, possibly, subsequent exhaustion of CD8 T cells in severe cases [152]. The description of three immune T-cell phenotypes identified in hospitalized patients might reflect the heterogeneity of patient response to SARS-CoV-2 infection [153].

An immunological and transcriptional analysis revealed a perturbed regulatory T-cell phenotype in severe COVID-19 patients with overexpression of various suppressive effectors and proinflammatory mediators [154]. Another study reported that the presence of regulatory T cells increased during mild-to-severe phases of infection and declined toward the more critical phase of COVID-19 infection [155]. This phenomenon is consistent with previously reported findings that a decrease in regulatory T cells was related to disease severity [156]. Thus, regulatory T cells are fundamental for immunological tolerance, and evidence suggests that reduced regulatory T-cell levels in severe COVID-19 might play a role in immune system dysregulation and lung injury [157].

Analysis of brains of patients who died with COVID-19 reveals an anatomical compartmentalization of the altered brain immune responses characterized by microgliosis, the formation of microglial nodules and specific perivascular CD8 T-cell clusters [158]. Cellular analysis of these microglial nodules showed significant T-cell–microglial crosstalk in association with increased immune activation in the rest of the tissue, suggesting an important role of microglial nodules in orchestrating neuroinflammation [158].

A compartmentalized T-cell response to CNS antigen is also supported by the analysis of T-cell receptor sequences in CSF and blood [159]. This aspect revealed clonal expansion of unique but unshared CD4 T-cell clones in COVID-19 CSF [159]. Additionally, the discovery of COVID-19-associated transcriptional changes in T cells in the CSF suggests cell–cell interactions unique to the CSF of infected patients [159]. Furthermore, an intriguing study showed that CD8 T lymphocytes and CD68 macrophages generated inflammatory perivascular infiltrates in some brain regions, supporting the idea of a link between microvascular and neurological injuries in SARS-CoV-2 infection [160].

A study of 499 COVID-19 patients reported that 95% of patients in the ICU group exhibited a decrease in both CD4/CD8 T cells, in addition to high expression of PD-1 and Tim-3 exhaustion markers on their T cells [161]. Moreover, PD-1 was high on CD8 T cells in microglial nodules or in T cells at the vasculature, suggesting that these cells are likely important mediators of neuroinflammation and BBB leakage in SARS-CoV-2 infection [158]. Surprisingly, a study demonstrated that CD8 T cells from acute and convalescent COVID-19 patients, which express PD1, were not exhausted but functional and activated [162]. Another study reported no significant evidence of CD4/CD8 T-cell exhaustion and no substantial expression of proinflammatory cytokine genes by peripheral monocytes, T or NK cells [163]. However, single-cell RNA sequencing demonstrated that the presence of myeloid cells increased, whereas the presence of NK and T cells decreased in the peripheral blood of COVID-19 patients, and that immune cell compositions differed between patients in early and late recovery stages [164].

An additional proposed pattern of immune dysregulation in severe COVID-19 is characterized by excessive release of IL6, sustained inflammation and profound lymphopenia [165]. However, whether steroid use during hospitalization plays a role in determining lymphopenia requires further investigation [166].

The analysis of post-mortem COVID-19 brains identified T-cell lymphocytic perivascular and meningeal infiltrate, supporting a pronounced CNS involvement [167]. In addition, a study of a cohort of cancer patients with neurologic manifestation of COVID-19 described the presence of a wide range of intracranial inflammatory cytokines [168].

Histopathological examination focused on the olfactory bulb and brainstem of patients who died from COVID-19 revealed the presence of T cells in the perivascular spaces [169]. Moreover, activated microglia were found close to neurons in the olfactory bulb, substantia nigra, dorsal motor nucleus of the vagal nerve and in the pre-Bötzinger complex in the medulla, which is involved in the generation of spontaneous rhythmic breathing [169]. These findings highlight the potential connections between the nervous system and the lungs in the pathogenicity of SARS-CoV-2 disease progression.

## 5. Age-Related Immune System Changes and COVID-19

Age-associated decline of the immune system makes older people more vulnerable to SARS-CoV-2 infection than younger patients. Evidence demonstrates that age is one of the most important risk factor for severe COVID-19 disease and adverse health outcomes [170,171]; for example, the pneumonia severity index score of the elderly group was higher than that of the young and middle-aged groups in [172,173].

In COVID-19 elderly patients, senescent cells may accumulate in the respiratory tract during aging, secrete proinflammatory mediators and promote inhibition of T-cell responses in infected cells [174].

Autophagy, which contributes to mitigation of inflammatory pathways, declines with aging [175]. Additionally, alveolar macrophages show impaired functions, such as diminished phagocytosis and altered antiviral response, whereas the increased number of neutrophils in the low respiratory tract plays a role in age-associated inflammation [176].

Inflammaging is an important hallmark of advanced age and refers to an aberrant systemic inflammation sustained by elevated levels of circulating and tissue proinflammatory cytokines coupled with a reduced ability to deal with immunological threats [176]. This phenomenon, which is associated with altered expression of ACE2, immune senescence, impaired autophagy and excessive ROS production, could be connected to the risk of cytokine storm in some older patients with severe COVID-19 [177].

Older people with comorbidities, including hypertension and diabetes, are more susceptible to severe COVID-19 and cerebrovascular events [178]. In addition, age-related changes impair the BBB, increase its permeability and further impact microglia housekeeping functions [179].

The production of important antiviral cytokines, such as type I INF, is impaired with progressing age [180]. The reduction in type I INF production, the increase in proinflammatory monocytes and the accumulation of functionally exhausted and senescent CD4/CD8 T cells is similar between the immune profiles of severe COVID-19 patients and healthy older adults [181]. In contrast, analysis of immune responses in 113 patients with COVID-19 showed no significative differences in the development of moderate or severe disease in association with age [129]. However, adaptive immunity is significantly altered with age, including skewed haematopoiesis toward myeloid production and suppression of lymphopoiesis, whereas T cells become progressively hypofunctional and hyporesponsive [176].

SARS-CoV-2 can also exacerbate the imbalance of an aged immune system, inducing a direct depletion of CD4 T cells [182]. A study of a group of elderly COVID-19 patients revealed that lymphocytopenia was detectable in 63.2% of cases, with a significant decrease in T-cell counts in patients who died from the infection [183].

Old age and chronic comorbidities adversely affect the outcome of COVID-19 patients [50]. Diabetes, obesity, cardiovascular disease, and lung and liver diseases are strongly associated with severe forms of SARS-CoV-2 infection [184,185,186]. These clinical conditions are common in older adults [187,188]. Moreover, an interesting study highlighted that risks of severe illness and death are highest for COVID-19 patients with anxiety, obesity and complicated diabetes [189].

Severe neurological complications were consistently present and indicative of negative clinical outcomes in elderly patients with COVID-19 [190]. A recent cohort study showed that old age, comorbidities and neurological manifestations were more common in hospitalized COVID-19 patients compared to non-hospitalized patients [191].

**Figure 2:**
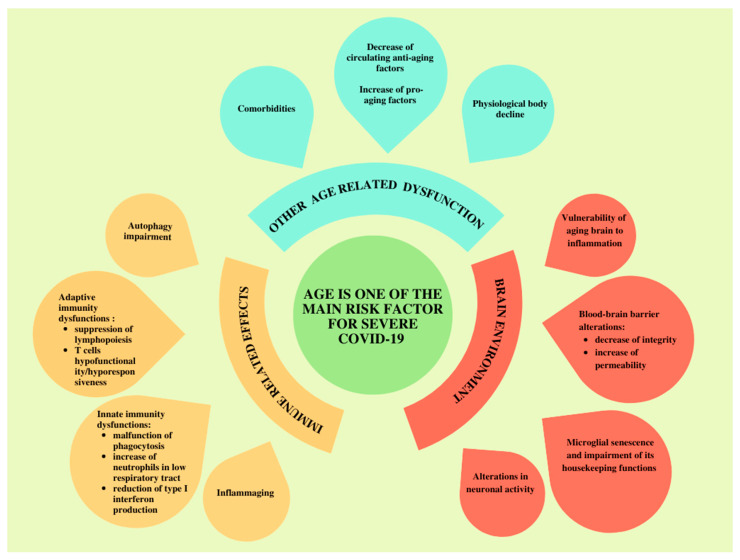
Aging and severe COVID-19: possible roles of immune system effects, brain environment and other age-related dysfunctions. Age is one of the most important risk factors for severe forms of infection [170,171]. Each of age-related immune system changes [175,176,181] reported in yellow on the left side of the figure could contribute to the dysregulation of immune responses against the virus. In addition, comorbidities [184] and other age-related dysfunction can contribute to the risk of developing severe forms of SARS-CoV-2 infection, as shown in blue at the top of the figure. The effects of aging on the brain environment are reported in red on the right side of Figure 2, including alterations in neuronal and microglial activities and dysfunction of the blood–brain barrier [179,193]. Thus, age-related immunity dysregulations and brain environment alterations might play a role in the pathogenicity of disease, supporting neurological involvement in severe COVID-19 infection.

Based on these findings, we propose a model in which the physiological body decline, comorbidities and age-associated changes in the immune system may contribute to disease severity and facilitate neurological involvement during SARS-CoV-2 infection (Figure 2).

The decrease in circulating antiaging factors and the increase in pro-aging factors influence microglial senescence, underlying the important communication between the systemic environment and brain aging [192].

The aging brain is also vulnerable to inflammation and proinflammatory factors in the circulatory system, which can promote cognitive decline [193].

Aging is associated with alterations in neuronal activity and changes in neuropeptide release in the lung mucosa, which could lead to immunological modulation and aberrant pulmonary function during SARS-CoV-2 infection [125]. However, the lack of animal models to define the connection between aging and COVID-19 infection limits the current knowledge of age-related host immunity responses against the virus [180]. Nevertheless, communication between the CNS and the immune system is important for brain homeostasis and function and depends on the integrity of both systems [194]. Thus, impaired neuro–immune crosstalk related to age might contribute to severe clinical disease progression in COVID-19 patients.

## 6. Therapy

Various therapeutic strategies are currently under investigation, with the aim of fighting severe SARS-CoV-2 infection. Antiviral drugs, steroids and monoclonal antibodies are widely used, with varying results with respect to patient outcomes. However, there is a lack of studies exploring the effects of COVID-19 therapies on neurologic symptoms.

The use of monoclonal antibodies against specific cytokines combined with corticosteroid could be useful in preventing cytokine storm, thereby promoting an improvement in clinical outcomes in severe COVID-19 patients [195]. Corticosteroids provide clinical benefits and reduce mortality in critically ill COVID-19 patients who receive respiratory support with oxygen or mechanical ventilation [196,197]. ICU patients with COVID-19-related encephalopathy responded to high-dose steroid therapy [198]. Moreover, a clinical trial comparing treatments with respect to the outcome of 28-day mortality showed that dexamethasone was associated with a reduction in 28-day mortality in hospitalized COVID-19 patients receiving respiratory support [199]. In contrast, a meta-analysis showed that steroid therapy did not reduce the mortality risk and the period of viral shedding, which were the two main outcomes of the study [200]. Thus, the use of steroids against SARS-CoV-2 infection and their efficacy remain still controversial.

Treatment with intravenous immunoglobulin therapy, which has anti-cytokine and anti-inflammatory effects, resulted in significant improvement in neurological symptoms in COVID-19 patients [201].

A case study of COVID-19 patients with autoimmune encephalitis reported a strong improvement following plasmapheresis both in a clinical setting and according to laboratory findings [202].

Although monoclonal antibody treatment for SARS-CoV-2 infection has focused on the respiratory tract, its beneficial effects and anti-inflammatory properties with respect to COVID-19 neurologic symptoms require further investigation [203].

Tocilizumab is one of the first monoclonal antibodies used against SARS-CoV-2 infection, although it has poor penetration into the CNS, and data on its benefits are limited [204]. Critically ill patients with COVID-19 treated with tocilizumab within the first 2 days of ICU admission showed reduced risk of death compared to untreated patients [205]. The JAK inhibitor baricitinib has better brain uptake and could decrease viral titre, IL6 levels and symptoms, such as fever and cough, likely owing to its antiviral and anti-inflammatory activities [132,206,207].

Another important aspect to consider is that neurologic symptoms may be manifestations of side effects of treatments against COVID-19. For example, ribavirin and interferon alpha result in both neuropathic and neuropsychiatric sequelae [208], whereas antiviral drugs may induce seizures [209].

Considering that ACE2 is the main receptor for SARS-CoV-2 cell infection, the renin-angiotensin system is likely a suitable target for therapeutic strategies.

In a multicentre, retrospective study, the use of ACE inhibitors was associated with improved prognosis, whereas angiotensin II receptor blockers were associated with worse outcome, especially in hypertensive COVID-19 elderly patients [210]. In contrast, other evidence shows that RAS inhibitors did not represent a risk factor, and withdrawal from these drugs made patients with unstable clinical status more vulnerable to early risks [211,212].

Treatment with human recombinant soluble ACE2 (hrsACE2) was strongly active in cleaving angiotensin II in angiotensin 1–7, and a concurrent reduction in inflammatory cytokines was observed in a severe case of COVID-19 [213]. An intriguing study demonstrated that hrsACE2 markedly inhibited SARS-CoV-2 infection in engineered human capillary organoids [214]. Based on these findings, additional studies investigating the involvement of the vasculature in infection spread are needed.

Various therapeutic approaches are available, with the goal of improving the adaptive immune system response against SARS-CoV-2 infection. Late therapeutic interventions with blockers of both PD-1 and IL6 pathways improved clinical outcomes of severe COVID-19 cases and reversed T-cell exhaustion, reducing tissues damage and hyperinflammatory consequences [215]. COVID-19 patients who have tachypnoea, hypoxia and saturation <90% require oxygen therapy and other respiratory therapies, including mechanical ventilation [216]. However, the role of hypoxia and the effects of respiratory therapies on the nervous system in patients infected by SARS-CoV-2 need to be elucidated.

In addition, proinflammatory cytokines and hypoxia contribute to the development of coagulopathy [217], which represents a marker of poor prognosis in COVID-19 patients. Thus, anticoagulant treatment plays a key role in the management of the disease [217]. A prophylactic dose of low-molecular-weight heparin should be considered in all hospitalized COVID-19 patient [218], whereas direct oral anticoagulant use was not associated with reduced risk of severe forms of infection [219].

The oral antiviral agent molnupiravir, which was originally designed for other viruses, is a promising drug for treatment for SARS-CoV-2 infection [220]. Molnupiravir induces RNA mutagenesis by the viral-RNA-dependent RNA polymerase and interferes with viral replication and the formation of intact new viruses [221]. As recently described, the possible mutagenic activity of this drug against host cells should not be ignored and requires further investigation [222]. However, an interesting study on molnupiravir effects showed a strong reduction in virus titre in human lung tissue after two days of treatment post SARS-CoV-2 exposure [223]. Moreover, molnupiravir reduced the risk of hospitalization or death by approximately 50% in adult patients with mild-to-moderate COVID-19 and presented consistent efficacy across viral variants Gamma, Delta and Mu [224].

A randomized, placebo-controlled, double-blind phase 2/3 trial evaluating the efficacy of molnupiravir in hospitalized patients with COVID-19 showed that molnupiravir was not associated with clinical benefit [225]. On the other hand, a randomized, placebo-controlled, double-blind phase 3 trial revealed that early treatment with molnupiravir reduced the risk of hospitalization or death in non-hospitalized, unvaccinated adults with mild-to-moderate COVID-19 and at least one risk factor for severe COVID-19 [226]. Another trial reported that patients treated with molnupiravir showed faster normalization of CRP and oxygen saturation, and those hospitalized presented with fewer respiratory interventions and earlier discharge compared to the placebo group [227]. Based on these data, molnupiravir might reduce the risk of developing severe SARS-CoV-2 infection rather than treating severe COVID-19 illness. A possible explanation is that treatment with this antiviral drug is effective mainly during the early phases of infection, near the time of peak viral replication [225]. These encouraging results highlight the possibility for future studies on molnupiravir efficacy in COVID-19 patients.

## 7. Conclusions

The incidence of patients with severe COVID-19 infection presenting with neurological symptoms has continuously increased during the pandemic. Current findings related to immune system alterations, respiratory damage and neurological manifestations during SARS-CoV-2 infection support the hypothesis of a correlation between dysregulated neuro–immune interactions and severe clinical outcomes.

The dysregulation of both RAS and immune systems, as well as age-related decline of body functions, could contribute to disease progression, exposing elderly patients to adverse health outcomes. Conversely, a healthy balance of inflammatory responses (type I IFN), particularly at the initial phase of infection, and a coordinated adaptive immune response could prevent severe COVID-19.

Evidence shows increased risk of neurological disorders within 6 months following COVID-19 infection, with greater risks in critical patients necessitating hospitalization and intensive therapy unit admission, although not limited to these groups [228]. These findings provide novel perspectives on nervous system involvement during and after SARS-CoV-2 infection.

In the present review, we described various therapeutic strategies currently in use for the treatment of COVID-19; however, data on the effects of these therapies on neurological symptoms are limited. Thus, this review is meant to stimulate future studies to shed light on the complexity of SARS-CoV-2 pathogenesis.

## Figures and Tables

**Table 1:** Studies of neurological manifestations, laboratory and imaging findings associated with SARS-CoV-2 infection.

Study	Characteristics of the Study	SARS-CoV-2 Diagnostics	Age	Neurological Symptoms	Other Symptoms	Laboratory and Radiology Findings	Neurological Findings: CSF Analysis, Neuroimaging and Neurophysiology
Agarwal et al. [29]	404 patients hospitalized between 20 February 2020 and 4 May 2020.	All patients had laboratory-confirmed SARS-CoV-2 infection.	Median age: 61. Patients with neurological symptoms were older than those without neurological symptoms.	Neurological findings (*n* = 295). CNS symptoms (*n* = 208): altered mental status (*n* = 86) headache (*n* = 82) dizziness (*n* = 31). PNS symptoms (*n* = 163): myalgia (*n* = 131) impairment of taste (*n* = 27) impairment of smell (*n* = 18). Acute neurological manifestations (*n* = 99).	Group without neurological symptoms (*n* = 109): fever (*n* = 60) cough (*n* = 76) shortness of breath (*n* = 57). Group with neurological symptoms (*n* = 295): fever (*n* = 194) cough (*n* = 212) shortness of breath (*n* = 166).	Group with neurologic symptoms compared to group without neurological symptoms: lower absolute neutrophil count higher absolute lymphocyte count.	
Mao et al. [8]	Retrospective, observational case series of 214 hospitalized patients.	All patients had laboratory-confirmed SARS-CoV-2 infection.	Median age: 52.7. Patients with severe infection were older than those with nonsevere infection.	Neurological manifestations (*n* = 78) were more common in severe infections than nonsevere infections. CNS symptoms (*n* = 53): dizziness (*n* = 36) headache (*n* = 28) impaired consciousness (*n* = 16). PNS symptoms (*n* = 19): taste impairment (*n* = 12) smell impairment (*n* = 11.) Acute cerebrovascular disease (*n* = 9).	Symptoms at onset of illness: fever (*n* = 132) cough (*n* = 107) anorexia (*n* = 68) diarrhoea (*n* = 41) throat pain (*n* = 31).	Patients with severe infection compared to those with nonsevere infection: increased inflammatory response higher white blood cell count and neutrophil counts lower lymphocyte counts increased C-reactive protein (CRP) level. Patients with CNS symptoms compared to those without CNS symptoms: lower lymphocyte levels lower platelet counts. Lung CT: ground-glass opacities.	Brain CT: new onset of ischaemic stroke in a COVID-19 patient.
Varatharaj et al. [30]	Surveillance study of 153 cases collected through online network. Complete clinical datasets were available for 125 of 153 patients.	Among 125 patients, SARS-CoV-2 infection was: confirmed (*n* = 114) probable (*n* = 5) possible (*n* = 5).	Median age: 71 Altered mental status and cerebrovascular events affected older patients more than younger patients.	Referred to 125 patients: cerebrovascular events (*n* = 77) altered mental status (*n* = 39).			
Paterson et al. [31]	Retrospective study of neurological disorders in 43 patients referred to the National Hospital, Queen Square COVID-19 multidisciplinary team meeting.	SARS-CoV-2 PCR test: confirmed (*n*= 29) probable (*n* = 8) possible (*n* = 6).	Ages 16–85 years. Patients with encephalopathies were mostly older than 50 years old.	encephalopathy (*n* = 10) inflammatory CNS syndromes (*n* = 12) strokes (*n* = 8) peripheral syndromes (*n* = 8).		D-dimer > 7000 mg/L in patients who developed ischaemic stroke. CT pulmonary angiogram: pulmonary embolism in a stroke patient. Chest CT: typical of COVID-19 pneumonitis.	CSF analysis: negative for SARS-CoV-2. EEG: normal. Brain CT/MRI: signs of stroke. MRI abnormalities were common in patients with neuroinflammatory diseases.
Romero-Sánchez et al. [32]	Retrospective observational study of 841 hospitalized patients, 329 of whom had severe COVID-19.	All patients had laboratory-confirmed SARS-CoV-2 infection.	Mean age: 66.4. Patients with severe disease were older than those with mild disease.	Neurological manifestations (*n* = 483): myalgia (*n* = 145) headache (*n* = 119) dizziness (*n* = 51) anosmia (*n* = 41) dysgeusia (*n* = 52) disorders of consciousness (*n* = 165) seizures (*n* = 6) cerebrovascular manifestation (*n* = 14).	Symptoms upon admission: fever (*n* = 736) cough (*n* = 644) dyspnoea (637).	Severe disease: elevated CK, ferritin, CRP and D-dimer lower lymphocyte count. Chest X-ray findings: bilateral pneumonia (*n* = 721).	CSF analysis: normal. EEG: moderate encephalopathy (*n* = 2). Brain MRI of a patient with multiple brain hemorraghes: pattern resembling posterior reversible encephalopathy syndrome. Brain MRI of a patient with encephalitis: bitemporal lobe involvement.
Zanin et al. [34]	Case report of a COVID-19 patient admitted for interstitial pneumonia and seizures.	RT-PCR test positive for SARS-CoV-2.	54 years old.	Anosmia, ageusia and two seizures.	Interstitial pneumonia.	Moderate lymphocytopenia with mild elevation of inflammatory indices. Chest X-ray: interstitial pneumonia.	CSF analysis: normal. EEG: two seizures starting from right frontotemporal region and diffusing in homologous contralateral hemisphere. Brain MRI: demyelinating lesions.
Mahammedi et al. [35]	Multicenter retrospective observational study of 725 hospitalized COVID-19 patients.	All patients had laboratory-confirmed SARS-CoV-2 infection.	Mean age: 69. Statistically significant association between altered mental status and patient age.	Acute neurologic symptoms (*n* = 108): altered mental status (*n* = 64) ischaemic stroke (*n* = 34) headache (*n* = 13) myalgias (*n* = 13) seizures (*n* = 10).			Brain MRI/CT: acute neuroimaging abnormalities (*n* = 51): ischaemic infarcts (*n* = 34) intracranial haemorrhages (*n* = 6).
Kremer et al. [36]	Retrospective multicenter study of 64 patients with COVID-19 and neurologic manifestations.	All patients had laboratory-confirmed SARS-CoV-2 infection.	Median age: 66.	confusion (*n* = 34) impaired consciousness (*n* = 25) clinical signs of corticospinal tract involvement (*n* = 20) agitation (*n* = 20) headache (*n* = 10).	ARDS (*n* = 33).		CSF analysis: negative for SARS-CoV-2. Brain MRI: abnormal (*n* = 36): ischaemic stroke (27%) leptomeningeal enhancement (17%) encephalitis (13%).
Kremer et al. [37]	Retrospective observational study of 37 patients evaluated from 23 March 2020 to 27 April 2020 at 16 hospitals.	All patients had laboratory-confirmed SARS-CoV-2 infection.	Mean age: 61.	alteration of consciousness (*n* = 27) confusion (*n* = 12) agitation (*n* = 7) seizures (*n* = 5) headache (*n* = 4) clinical signs of corticospinal tract involvement (*n* = 4).	ICU admission for acute respiratory failure (*n* = 32).	leukocytosis, lymphopenia, anaemia, elevated serum levels of CRP, ferritin, alanine aminotransferase, aspartate aminotransferase, urea, creatinine, fibrinogen and D-dimers.	CSF analysis: positive for SARS-CoV-2 RNA (*n* = 1). EEG (*n* = 26): normal (*n* = 2) realized under sedation (*n* = 6) nonspecific findings (*n* = 10) encephalopathy (*n* = 7) case of seizures (*n* = 1). Brain MRI: signal abnormalities in the medial temporal lobe (*n* = 16) nonconfluent multifocal lesions associated with haemorrhagic lesions (*n* = 11) extensive and isolated white matter microhaemorrhages (*n* = 9).
Paniz-Mondolfi et al. [38]	Case report of a 74-year-old male.	RT-PCR test positive for SARS-CoV-2.	74 years old.	Confusion upon admission.	Fever upon admission. Episodes of hypotension and progressively worsening SpO2. He continued to decompensate clinically and expired on day 11.	Thrombocytopenia and elevated inflammatory markers. Chest radiography: left basilar densities consistent with left lower lobe consolidation and a component of pleural fluid; right basilar densities and patchy densities in the right midlung.	CSF analysis: negative for SARS-CoV-2. Brain postmortem examination: the virus was detected in neural and capillary endothelial cells.
Filatov et al. [39]	Case report of a 74-year-old male.	RT-PCR test positive for SARS-CoV-2.	74 years old.	Headache and altered mental status.	Fever and cough upon admission. He developed respiratory failure requiring intubation, and he was transferred to ICU.	Chest X-ray: small right pleural effusion with bilateral ground glass opacities. Chest CT: patchy bibasilar consolidations and subpleural opacities.	CSF analysis: no evidence of CNS infection. EEG: bilateral slowing and focal slowing in the left temporal region with sharply contoured waves. CT scans of the head: no acute abnormalities.
Travi et al. [40]	Retrospective analysis of 901 patients admitted to hospital.	All patients had laboratory-confirmed SARS-CoV-2 infection.	Median age: 64.	Presence of at least one neurological symptom at admittance (*n* = 272): mental confusion (*n* = 61) stroke (*n* = 53) dysgeusia/anosmia (*n* = 82) seizure (*n* = 19) syncope (*n* = 81) headache (*n* = 39). The presence of any neurologic involvement was higher among those with moderate disease compared to those with severe or critical disease.	Only respiratory symptoms (*n*= 629). Respiratory/neurologic symptoms (*n* = 111).		CSF analysis: SARS-CoV-2 was detected only in one patient. The other patients had typical inflammatory CSF abnormalities.

## Data Availability

Not applicable.
